# A Machine Learning Approach to Diagnosing Lung and Colon Cancer Using a Deep Learning-Based Classification Framework

**DOI:** 10.3390/s21030748

**Published:** 2021-01-22

**Authors:** Mehedi Masud, Niloy Sikder, Abdullah-Al Nahid, Anupam Kumar Bairagi, Mohammed A. AlZain

**Affiliations:** 1 Department of Computer Science, College of Computers and Information Technology, Taif University, P.O. Box 11099, Taif 21944, Saudi Arabia; 2 Computer Science and Engineering Discipline, Khulna University, Khulna 9208, Bangladesh; niloysikder333@gmail.com (N.S.); anupam@ku.ac.bd (A.K.B.); 3 Electronics and Communication Engineering Discipline, Khulna University, Khulna 9208, Bangladesh; nahid.ece.ku@ku.ac.bd; 4 Department of Information Technology, College of Computers and Information Technology, Taif University, P.O. Box 11099, Taif 21944, Saudi Arabia; m.alzain@tu.edu.sa

**Keywords:** deep learning, lung cancer detection, colon cancer detection, histopathological image analysis, image classification

## Abstract

The field of Medicine and Healthcare has attained revolutionary advancements in the last forty years. Within this period, the actual reasons behind numerous diseases were unveiled, novel diagnostic methods were designed, and new medicines were developed. Even after all these achievements, diseases like cancer continue to haunt us since we are still vulnerable to them. Cancer is the second leading cause of death globally; about one in every six people die suffering from it. Among many types of cancers, the lung and colon variants are the most common and deadliest ones. Together, they account for more than 25% of all cancer cases. However, identifying the disease at an early stage significantly improves the chances of survival. Cancer diagnosis can be automated by using the potential of Artificial Intelligence (AI), which allows us to assess more cases in less time and cost. With the help of modern Deep Learning (DL) and Digital Image Processing (DIP) techniques, this paper inscribes a classification framework to differentiate among five types of lung and colon tissues (two benign and three malignant) by analyzing their histopathological images. The acquired results show that the proposed framework can identify cancer tissues with a maximum of 96.33% accuracy. Implementation of this model will help medical professionals to develop an automatic and reliable system capable of identifying various types of lung and colon cancers.

## 1. Introduction

Cancer refers to a range of diseases where abnormal cells develop inside the human body because of random mutations. Upon generation, these cells divide uncontrollably and spread throughout the organs. If it goes untreated, most types of cancer can eventually result in death. Cancer is the principal cause of death worldwide after cardiovascular diseases. In 2018, more than 18 million new cancer cases were reported worldwide, along with 9.55 million deaths [1]. According to the predictions made by the American Cancer Society, more than 1.8 million new cancer cases will be reported in 2020, and over 606,000 death will occur in the USA alone [2]. Based on the data collected between 2015 and 2017, two out of every five American citizens will be diagnosed with cancer at some point in their lifetime. Cancer cells can develop in any part of the body; the most common organs affected are lungs, breasts, brain, colon, rectum, liver, stomach, skin, and prostate. Among them, lung and colon cancers result in the greatest number of deaths both in men and women. In 2018, they were responsible for 2.9 million new cases, along with over 2.5 million deaths in the USA alone [3]. There are many reasons behind cancer, ranging from behavioral traits such as high body mass index, tobacco and alcohol usage to physical carcinogens, such as exposure to ultraviolet rays and radiation, including certain biological and genetic carcinogens [3]. However, the cause may vary from one patient to another. Common cancer symptoms are pain, fatigue, nausea, persistent cough, breathing difficulties, weight loss, muscle pain, bleeding, bruising, and many more [4]. Then again, neither of these symptoms are exclusive to cancer, nor all of them are apparent in every patient. As a result, it is hard to determine the presence of cancer without a thorough diagnostic procedure such as Computed Tomography (CT) scan, Magnetic Resonance Imaging (MRI), Positron Emission Tomography (PET) scan, ultrasound, or Biopsy. In many cases, the victims show little to no symptom at the early stages; and when symptoms become apparent, more often than not, it is already too late.

In some cases, a person inherits the abnormal gene that leads to cancer from his/her parents. People who carry the risk of developing inherited cancers need to go through regular checkups. These diagnostic methods are costly, and many people cannot afford them. Approximately 70% of deaths due to cancer occur in low and middle-income countries [3]. According to the data collected in 2016, only 26% of countries with low incomes had the pathological services necessary to diagnose cancer available to the public; countries with high incomes, on the other hand, could offer diagnosis and treatment services to over 90% of their population [3]. Not just cancer, the lack of proper diagnosis leaves the people of developing and underdeveloped countries extremely vulnerable to other diseases as well. To overcome this challenge, these countries have to invest heavily in the public health sector, set up numerous laboratories and pathology centers with the necessary equipment, and train more individuals to carry out the diagnostic procedures. Furthermore, they have to keep the charges of these tests within reach of the people who live below the poverty line. Admittedly, these goals are difficult to achieve for any such country in the world, and even if it is possible, all of these things will not happen overnight. If we want to stay in the fight against cancer and give these people a realistic chance of survival, we have to look for alternative ways of diagnosis.

According to the Tumor-Node-Metastasis (TNM) system developed and maintained by the American Joint Committee on Cancer (AJCC), most of the cancers have five different stages—0, Stage I, Stage II, Stage III, and Stage IV [5]. The system takes into account numerous factors, including the size and location of the primary tumor, the extent of its spread to the lymph nodes and other organs, and the presence of any biomarkers that influence the spread of cancer. The chances of survival vary significantly at different stages. For example, in the case of colon cancer, more than 93% of people within the age bracket of 18 and 65 years can survive with proper treatment if they are diagnosed at Stage 0; whereas, the survival rates at the later three stages are 87%, 74%, and 18%, respectively [6]. For colon cancer patients, the chance of survival falls from 70% at Stage 0 to a frightening 13% at Stage IV. As discussed before, we do not have a guaranteed treatment for cancer yet, which means the earlier a person is diagnosed, the more time doctors have to devise a treatment plan for him/her, and the patient stands a better chance to beat the disease. Early diagnosis and proper treatment are currently the only way to reduce the number of deaths due to cancer [7]. However, most of the population is deprived of proper diagnostic facilities, which makes the battle against this virulent disease even more unamenable.

Surprisingly, a possible solution to this problem has come from a field quite dissimilar to Medicine and Healthcare. Computer Science has probably advanced the most in the last fifty years compared to other branches of science and technology. Machine Learning (ML) has a wide range of applications in Pathology, ranging from the detection of diseases to intelligent systems, which, judging by the patients’ symptoms, can prescribe conventional medicines [8]. The latter area is still in its infancy, and a lot of research works need to be done before such applications can be relied on for clinical practices. Nonetheless, it ascertains AI’s power and implies how it will be used in the medical sector in the coming years.

AI has shown incredible potential in the sector of diagnosis and offered us a capable alternative to the traditional diagnostic methods. At present, the process of diagnosis of a particular disease requires taking samples from a patient, performing a set of tests on those samples, converting the results into an interpretable form, and finally, the involvement of a trained individual to make decisions based on those results. Now, if the samples collected from a patient are digital in nature or are digitalized by some means, we can use machines to analyze them. We can then provide them with a set of data containing decisions on similar cases encountered in the past. Finally, we can instruct them to identify the diseases present in the new patient. In ML, making decisions that rely on the knowledge gathered from previous scenarios is known as supervised learning. Numerous supervised learning algorithms have been developed in the last three decades, and they are very proficient in working with biomedical data.

ML algorithms have been used in the classification and prediction of various types of biomedical signals. The development of Deep Learning (DL) algorithms has enabled machines to process high-dimensional data such as image, multidimensional anatomical image, and video as well. DL is a sub-field of ML that describes the learning algorithms inspired by the structure and function of the human brain [9]. DL uses the power of Artificial Neural Networks (ANNs) to achieve enhanced pattern recognition abilities. A detailed overview of the numerous ML techniques used in various lung and colon cancer diagnoses is provided in the next section. Above all, it is apparent that AI has given the field of medical diagnosis a new dimension, and it is gradually becoming a viable substitute for the traditional diagnostic methods.

Nevertheless, AI is still a long way away from taking over the diagnostic sector. Even though AI models are promising on paper and in controlled experiments, they have not yet reached that level of reliability, where they can be trusted with the duty of making life-altering decisions. Surely, some simple diagnostic procedures are carried out solely by machines with very little to no human intervention. However, AI methods are often not accurate enough, and their performance in the practical situation remains in question. Furthermore, there are some ethical dilemmas as well. However, these challenges make this field more open to further research, which is very inviting to the researchers. And they are tackling these challenges by collecting more practical data, developing new and improved learning algorithms, and putting the resultant models through rigorous tests. In this paper, we describe the outcome of a similar effort. Using a new set of histopathological images, we have developed a Convolutional Neural Network (CNN)-based novel classification method to distinguish among five different types of lung and colon tissues. The results show that the model is well-capable of classifying the associated lung and colon cancer varieties with high reliability. Necessary graphs, tables, charts, and other illustrations have been provided for easy interpretation while describing this cancer diagnosis approach in the subsequent sections.

The rest of the paper is organized as follows. Section 2 provides an overview of the previous works similar to our effort. Section 3 provides an overview of the contents of the employed dataset. Section 4 elucidates the principles of the methods and techniques required to build this model. Section 5 outlines the experimental setup, presents its outcomes, and provides brief discussions where they are necessary. Finally, Section 6 gives a summary of the work described in this article, along with some scopes of further research.

## 2. Related Works

The potential of computers in medical diagnosis was first recognized by Lee Lusted in 1955 [10]. Eight years later, Lodwick et al. digitized chest X-rays for the first time to develop Computer-Aided Diagnosis (CAD) applications and applied them to diagnose Lung cancer [11]. Published in 1963, their research marks the first practical use of computers in medical image diagnosis [12]. Throughout the 70s and 80s, lung cancer identification using chest radiographs was one of the most researched CAD applications. However, the invention of the DL methods has changed the field altogether. Researchers have used both DL and non-DL based learning algorithms in almost all types of a cancer diagnosis. Since our work belongs to the lung and colon cancer diagnosis domain, we will elaborately discuss the reported methods in these two areas. These approaches vary in terms of the type of images used, the processing techniques applied to those images, the kind of features extracted, and the architecture of the ML model used for cancer identification [13]. In the next few paragraphs, we will describe some of the prominent studies published in recent years whose objectives are similar to ours.

In 2013, Shi et al. described a multi-modal Sparse Representation-based Classification (mSRC) method for lung cancer diagnosis [14]. Their study captured needle biopsy specimens and automatically segmented 4372 cell nuclei regions for lung cancer classification. Their method reached an 88.10% classification accuracy on average. Xu et al. worked with a set of histopathological colon images, extracted four types of features from them, and employed three types of Support Vector Machines (SVMs) for classification [15]. Instead of single-level classification, they carried out a multi-label classification to identify multiple types of cancer residing in different image areas.

In 2014, Kuruvilla and Gunavathi proposed a CAD method for lung cancer classification based on the analysis of CT scan images [16]. They extracted six types of statistical features from those images and classified them using two Artificial Neural Networks (ANNs), one with only forward propagation and the other one with forward and back-propagation. According to this elaborate study, skewness provides the best classification outcome when paired with ANN with back-propagation. Deppen et al. inscribed a noninvasive pulmonary nodule diagnosis method based on PET scans analysis along with Fludeoxyglucose F-18 (FDG) data [17]. They tested the method on 8511 cases, among which 60% were malignant, and their model detected them with high sensitivity and specificity.

In 2016, Sirinukunwattana et al. described a Spatially Constrained CNN (SC-CNN) method to detect and classify four nuclei types in colon cancers based on histopathological images [18]. Their proposed method does not require the segmentation of nuclei and can provide a maximum F-measure of 80.2% while classifying them. Kuepper et al. reported a label-free classification method for colon cancer grading [19]. In this study, they used infrared spectral histopathology images and different dedifferentiation states of colon carcinoma. The classification was performed by a Decision Tree (DT)-based supervised learning method called Random Forest (RF).

In 2017, Shen et al. described a Multi-crop CNN (MC-CNN)-based nodule malignancy classification method [20]. Their approach’s specialty is that they did not use any segmentation or feature extraction techniques on the CT scan images they worked with. They relied entirely on their ML model for the intended lung nodule detection and achieved an 87.14% classification accuracy. Sun et al. used CNN and Deep Belief Network (DBN) on 134,668 CT scan images to determine the presence of lung cancer in them [21]. Yuan et al. detailed a DL method that can detect polyps automatically from colonoscopy videos [22]. For classification, they used AlexNet, a well-known CNN-based architecture that resulted in a 91.47% classification accuracy.

In 2018, Selvanambi et al. provided a Lung cancer prediction method based on glowworm swarm optimization (GSO) using images from multiple sources [23]. They chose the Recurrent Neural Network (RNN) as their learning algorithm and obtained a maximum of 98% accuracy. De Carvalho Filho et al. proposed a CNN-based lung cancer identification method and tested it on a dataset containing over 50,500 CT scan images [24]. Da Nóbrega et al. described a method to identify the malignant nodule in the lung using ResNet50, a CNN-based learning algorithm [25]. Their study also explores other learning methods, including Transfer Learning, ImageNet, MobileNet, Xception, and InceptionV3. Masood et al. proposed a CAD method for pulmonary cancer detection and stage classification [26]. The authors used CNN and DFCNet in their study and tested their model on six different datasets. Babu et al. described an RF-based classification model to predict the presence of colon cancer by analyzing histopathological cancer images [27]. First, they took the R-G-B images to the HSV plane and then performed wavelet decomposition to extract features. By changing the level of image magnification, they achieved a maximum of 85.4% classification accuracy. Mo et al. used a method based on Faster R-CNN for colon cancer detection [28]. They used an approximate joint optimization that can optimize classification and regression losses at the same time. Urban et al. reported a method that can identify polyps from colonoscopy images with a 96% classification accuracy [29]. The authors hand-labeled 8641 colonoscopy images collected from 2000 patients and trained a CNN model with them. Then, they tested their method on 20 colonoscopy videos having a total duration of five hours. Akbari et al. proposed a CNN-based classification method with binarized weights to identify colorectal cancer from colonoscopy videos [30]. They tested their method using the data collected from the Asu Mayo Test clinic database and achieved over 90% classification accuracy.

In 2019, Shakeel et al. presented an automatic Lung cancer detection method based on CT scan images [31]. They applied bin smoothing normalization for image de-noising and selected features using the minimum repetition and Wolf heuristic feature selection process. The most interesting approach of this study is the classifier’s choice; the authors used a Discrete AdaBoost optimized ensemble learning generalized neural network (DAELGNN) and obtained over 99% classification accuracy. Toraman et al. reported a study whose aim was to classify colon cancer’s likelihood using Fourier Transform Infrared (FTIR) spectroscopy signals [32]. The authors extracted several statistical features from those signals and then used SVM and ANN to classify them, which resulted in a 95.71% classification accuracy (ANN).

In 2020, Suresh and Mohan described a lung cancer diagnosis method based on nodule region of interest (ROI)-based feature learning using CNN. They collected CT scan images from the Lung Image Database Consortium (LIDC) and Infectious Disease Research Institute (IDRI) databases and employed Generative Adversarial Networks (GANs) to generate additional images to increase the sample size. They achieved a 93.9% classification accuracy (maximum) using CNN-based classification algorithms [33]. Masud et al. described a pulmonary nodule detection method based on CT scan images using a light CNN architecture [34]. Tested on the LIDC dataset, their model achieved a 97.9% classification accuracy while distinguishing among normal, benign, and malignant cases. Shakeel et al. proposed another CT scan image-based lung cancer detection method. Upon removing noise from the images, they employed an Improved Deep Neural Network (IDNN) for image segmentation and various Ensemble Methods (EM) for image classification [35].

## 3. Lung and Colon Cancer Dataset

This research worked with a new (published in 2020) lung and colon cancer histopathological image dataset known as the LC25000 dataset [36]. Assembled by Andrew A. Borkowski and his associates, this dataset contains 25,000 color-images of five types of lung and colon tissues [37]. These variants are Colon Adenocarcinoma, Benign Colonic Tissue, Lung Adenocarcinoma, Benign Lung Tissue, and Lung Squamous Cell Carcinoma. Colon Adenocarcinoma is the most common type of colon cancer, which makes up more than 95% of all colon cancer cases. Adenocarcinoma occurs when a particular type of polyp (tissue growth) called Adenoma is developed inside the large intestine, which later turns into cancer. Lung Adenocarcinoma accounts for about 40% of all lung cancers and is found more in women than men. This type of cancer cells usually develops in the glandular cells, and then spread towards the alveoli inside the lungs. All the tumors developed in the lungs and colon are not cancerous, as they do not spread to other parts of the body. These types of tumors are called benign tumors, which are usually not life-threatening. However, they still need to be surgically removed and checked for the presence of cancer through Biopsy. And finally, Lung Squamous Cell Carcinoma is a kind of small-cell cancer that develops in the lungs’ air passages or bronchi. It is the second most common type of lung cancer and accounts for about 30% of all cases.

**Figure 1:**
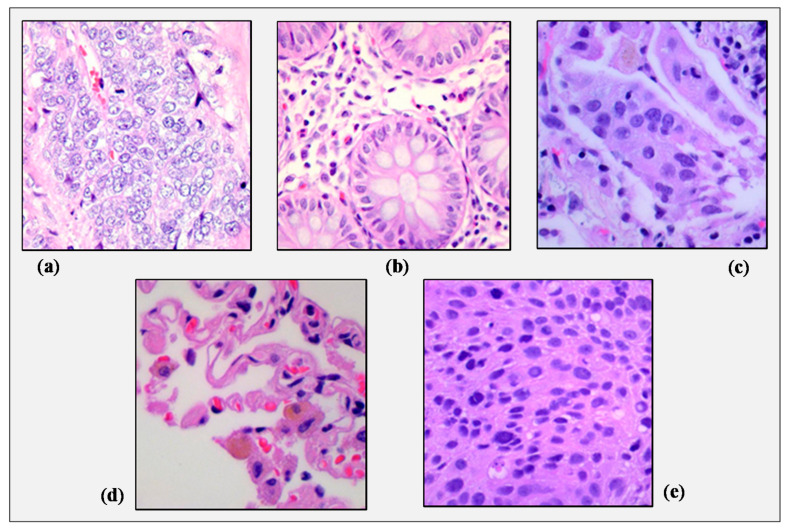
Sample images of (**a**) colon adenocarcinoma, (**b**) colon benign tissue, (**c**) lung adenocarcinoma, (**d**) lung benign tissue, and (**e**) lung squamous cell carcinoma collected from the LC25000 dataset.

The LC25000 dataset images were collected at James A. Haley Veterans’ Hospital situated in Tampa, Florida. The authors primarily collected 1250 images (250 images of each type) of cancer tissues from pathology glass slides. They used image augmentation techniques to rotate and flip the original images under different conditions and thus, expanded the dataset to 25,000 images (5000 images in each class). The original images’ size was 1024 × 768 pixels, but before applying the augmentation techniques, they were cropped to 768 × 768 pixels to make them square. All the images present in the dataset are the Health Insurance Portability and Accountability Act (HIPAA) compliant, validated, and free to use. Figure 1 presents sample histopathological images of these five classes collected from the LC25000 dataset. Table 1 lists the contents of the dataset and the assigned class names and IDs used in Section 4.

## 4. Methodology

After choosing the histopathological image dataset, features were extracted from them using two separate algorithms. The collected features were concatenated to formulate a combined feature set, and classification was performed based on it using a multi-channel CNN [38]. These steps have been described in the following subsections.

### 4.1. Image Sharpening Using Unsharp Masking

Image preprocessing is essential to remove noise, enhance specific properties, and draw out useful information from images—making them more suitable for the learning algorithm. This research used two image transformation techniques: two-dimensional Discrete Fourier transform (2D-DFT) and Single-level discrete two-dimensional wavelet transform (2D-DWT) extract features from the histopathological cancer images. Prior to feature extraction, each image’s contrast was enhanced using a popular image sharpening method known as Unsharp Masking (UM). UM’s basic idea is to subtract a blurred version of the original image from the image itself. UM enhances the contrast where different colors meet each other and, thus, sharpens the original image. If we consider $I\left(m,n\right)$ as a sample image where m is the height and n is the image’s width (in pixels). However, since we are working with square images, in our case, m=n. The sharpened form of I, $I_{s}$ will be as follows:

(1) $$I_{s}\left(m,m\right)=I\left(m,m\right)+\lambda \nabla \left(m,m\right)$$

where, λ is a factor that adjusts the intensity of the correction (λ>0), and $\nabla \left(m,m\right)$ is a suitably-defined gradient at $\left(m,m\right)$ [39]. A variety of gradient function can be used for UM. The one that is most commonly used is called the discrete Laplacian operator, which is defined as [40]:

(2) $$\nabla \left(m,m\right)≜I\left(m,m\right)-\frac{1}{4}\left[I\left(m-1,m\right)+I\left(m,m-1\right)+I\left(m+1,m\right)+I\left(m,m+1\right)\right]$$

**Figure 2:**
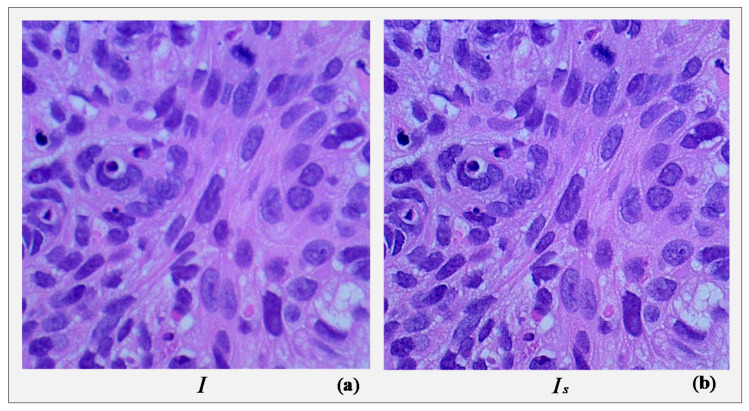
A sample colon cancer image (**a**) raw and (**b**) sharpened using unsharp masking.

We can get the desired $I_{s}$ from Equations (1) and (2). A UM outcome is influenced by three factors—radius, amount, and threshold of the operation. Radius governs the size of the area around the edges that is affected by sharpening. We need a large radius to sharpen wider regions around the edges and a smaller value for sharpening the narrower regions. Amount determines the strength of the sharpening effect. Threshold allows us to regulate whether or not a pixel will be considered as an edge pixel. It is also useful to reduce sharpening noise. In our case, we used [2, 2, 0.1] as the value of the radius, amount, and threshold, respectively. To sharpen an R-G-B image, it is primarily converted to “Lab” color space, then UM is applied only on the L-channel (lightness), and finally, the image is converted back to the R-G-B format. Figure 2 illustrates the result of image sharpening using UM on a sample histopathological image under the described conditions.

### 4.2. Feature Extraction from Cancer Images

As stated earlier, we will use two basic Digital Image Processing (DIP) techniques to extract features from the cancer histopathological images. These methods are briefly described below:

#### 4.2.1. Extraction of 2D Fourier Features

FT’s basic idea is to decompose a signal into an infinite number of sine and cosine functions. It was discovered afterward that it is possible to obtain the amplitude of those sine and cosine functions as well. If these functions are expressed in a series by using an integral, it is called the Fourier series. Discrete Fourier Transform (DFT) is the type of FT commonly used today to process signals in computer-based applications. DFT can extract the frequency components of any time-series signals of any length. If we have a *N*-point time-domain signal $P_{x}$, its *N*-point DFT can be expressed as follows:

(3) $$FP_{u}=\frac{1}{N}\overset{N-1}{\underset{x=0}{\sum }}P_{x}e^{-j\left(\frac{2\pi xu}{N}\right)}$$

Equation (3) describes the DFT of a one-dimensional (1D) data (signal). Since we are working with images, which are 2D data, we need an extended version of DFT. 2D-DFT transforms the pixels of an image based on their 2D spatial locations, which are indexed as co-ordinates $\left(x,y\right)$. It extracts the horizontal and vertical spatial frequencies $\left(u,v\right)$ from an image. If we consider an image, I of m×m pixels, its 2D-DFT can be expressed as [41]:

(4) $$FP_{u,v}=\frac{1}{m}\frac{1}{m}\overset{m-1}{\underset{x=0}{\sum }}\overset{m-1}{\underset{y=0}{\sum }}P_{x,y}e^{-j2\pi \left(\frac{ux}{m}+\frac{vy}{m}\right)}$$

Equation (4) can also be separated to show how DFT is done in two different axes:

(5) $$FP_{u,v}=\frac{1}{m^{2}}\overset{m-1}{\underset{x=0}{\sum }}\left\{\overset{m-1}{\underset{y=0}{\sum }}P_{x,y}e^{-j\left(\frac{2\pi vy}{m}\right)}\right\}e^{-j\left(\frac{2\pi ux}{m}\right)}$$

**Figure 3:**
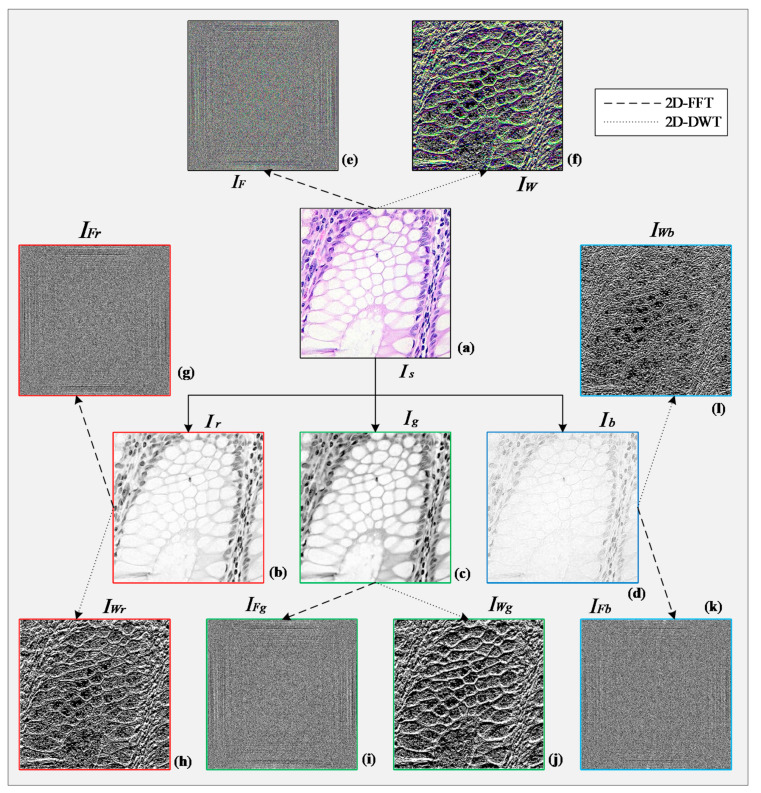
(**a**) A sharpened histopathological image; (**b**) red, (**c**) green, and (**d**) blue channel illustration of (**a**); (**e**) 2D-DFT and (**f**) 2D-DWT output of (**a**); (**g**) 2D-DFT and (**h**) 2D-DWT output of (**b**); (**i**) 2D-DFT and (**j**) 2D-DWT output of (**c**); and (**k**) 2D-DFT and (**l**) 2D-DWT output of (**d**).

2D-DFT highlights the lower frequency components of an image where most of the important information resides. On the other hand, the high-frequency components indicate the rapid changes in intensity, which typically occurs at the edges of objects [41]. Figure 3a shows a sample (sharpened) histopathological image. Figure 3b–d shows its red, green, and blue channel information, respectively. $I_{Fr}$, $I_{Fg},$ and $I_{Fb}$ are their corresponding 2D-DFT transformed form calculated using Equation (5) and illustrated in Figure 3g,i,k, respectively. Figure 3e is a combined representation of the previous three images that can be interpreted as the 2D-DFT of Figure 3a.

#### 4.2.2. Extraction of 2D Wavelet Features

**Figure 4:**
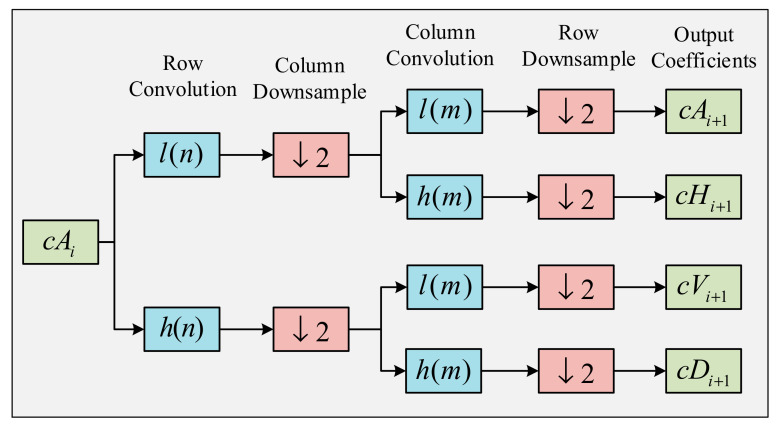
The process of decomposition in 2D-DWT.

Wavelets are mathematical functions that represent scaled and shifted forms of a signal. Wavelet transform (WT) is widely used for analyzing a signal into its frequency components at different resolutions. In the case of images, WT can reveal the frequency and spatial properties of that image at the same time. The Discrete wavelet transform (DWT) refers to any WT where the wavelets are discretely sampled. The first algorithm of DWT was developed by Alfréd Haar [42]. 1D-DFT can capture the frequency and location information of a signal simultaneously. It decomposes a signal into two separate components: approximation coefficients and detail coefficients, and raises the level. If we consider a *N*-point time-domain signal $P_{x}$, whose approximation and detail coefficients at level i are $A_{i}\left(x\right)$ and $D_{i}\left(x\right)$, respectively, then for the next level (i+1) values of these two coefficients would be as follows:

(6) $$A_{i+1}\left(x\right)=\overset{L-1}{\underset{k=0}{\sum }}h\left(k\right)A_{i}\left(2x-k\right)$$

(7) $$D_{i+1}\left(x\right)=\overset{L-1}{\underset{k=0}{\sum }}g\left(k\right)D_{i}\left(2x-k\right)$$

where, $h\left(k\right)$, $g\left(k\right)$, and L are the low-pass filter, high-pass filter, and the size of the filters [43]. In 2D-DWT, this operation is performed on both the rows and columns of an image. As a result, for images, four different matrices are level, namely, approximation coefficients (A), the horizontal detail coefficients (cH), vertical detail coefficients (cV), and diagonal detail coefficients (cD). If we take our previous image, I of m×m pixels, then these coefficients can be calculated as follows:

(8) $$cA_{i+1,\left(x,y\right)}=\frac{1}{2}\overset{m-l}{\underset{k=0}{\sum }}\overset{m-1}{\underset{l=0}{\sum }}d_{k}d_{l}cA_{i,\left(2x+k,2y+l\right)}$$

(9) $$cH_{i+1,\left(x,y\right)}=\frac{1}{2}\overset{m-l}{\underset{k=0}{\sum }}\overset{m-1}{\underset{l=0}{\sum }}b_{k}d_{l}cA_{i,\left(2x+k,2y+l\right)}$$

(10) $$cV_{i+1,\left(x,y\right)}=\frac{1}{2}\overset{m-l}{\underset{k=0}{\sum }}\overset{m-1}{\underset{l=0}{\sum }}d_{k}b_{l}cA_{i,\left(2x+k,2y+l\right)}$$

(11) $$cD_{i+1,\left(x,y\right)}=\frac{1}{2}\overset{m-l}{\underset{k=0}{\sum }}\overset{m-1}{\underset{l=0}{\sum }}b_{k}b_{l}cA_{i,\left(2x+k,2y+l\right)}$$

here, b and d are the scaling coefficients, k and l are the scaling coefficient indices, and x and y are the location indices [44]. Figure 4 illustrates how these four coefficients are calculated for a higher level (i+1), form the approximation coefficient of its previous level (i). Figure 3 represents the horizontal detail coefficients of Figure 3a extracted using 2D-DWT. $I_{Wr}$, $I_{Wg},$ and $I_{Wb}$ shows the information present in three separate channels calculated using Equation (8). In this study, we took only the horizontal detail coefficients into account and used them as a set of features drawn out from the original histopathological images.

#### 4.2.3. Feature Set Creation

**Figure 5:**
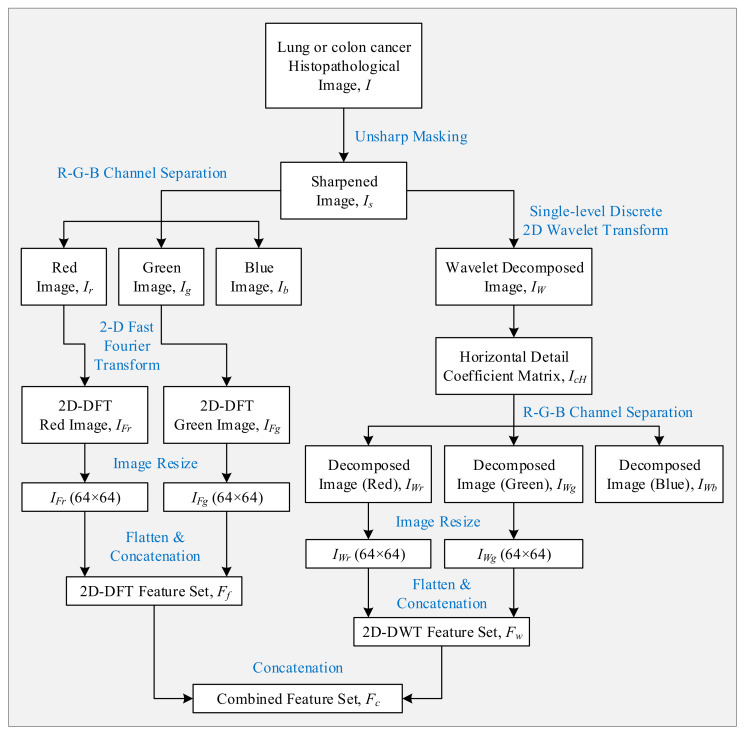
The workflow of the feature extraction and feature set creation process.

**Figure 6:**
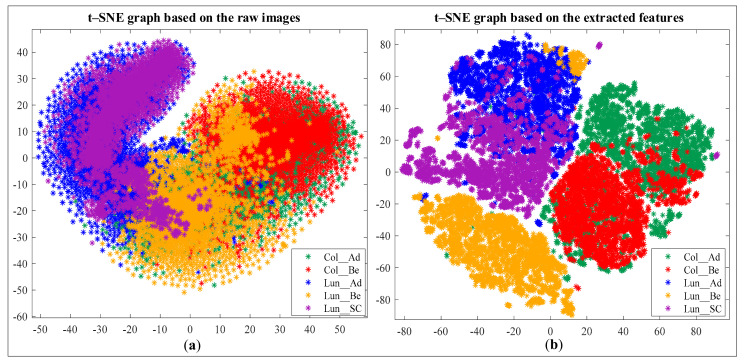
t-SNE graph of the dataset based on (**a**) the raw features and (**b**) the (combined) extracted features.

As seen in Figure 3b–d, the red and green channel of a histopathological image contains more distinctive information than the blue channel. That is why we will consider only the features extracted from the previous two channels and omit the last one. It will reduce the number of features, and in turn, reduce the complexity of the CNN model. The entire process of feature extraction, channel selection, and feature set creation has been portrayed sequentially in Figure 5. A resize operation was performed on the frequency-domain and wavelet-domain images to reduce their size to 64 × 64 pixels. Each image was then flattened to a row vector, and all the resultant vectors (25,000) were vertically concatenated to get the 2D-FFT feature and 2D-DWT feature subsets ($F_{f}$ and $F_{w}$). A further horizontal concatenation of these two subsets of features resulted in the combined feature set ($F_{c}$), which represents the samples of the dataset in the learning and classification stages. Figure 6a shows the t-Distributed Stochastic Neighbor Embedding (t-SNE) graph of the raw histopathological images (64 × 64 pixels) of the LC25000 dataset [45]. As the figure shows, samples of all the five classes are bounded very closely. Figure 6b shows the t-SNE graph of the samples based on the features we extracted following our algorithm. As can be seen, the samples of the *Col_Ad*, *Col_Be*, and *Lun_Be* formed individual and mostly separated clusters in the 2D plane. This implies that most learning algorithms will have a high classification rate of these classes. However, *Lung_Be* and *Lung_SC* samples are cramped together, meaning they will be harder to distinguish. A powerful learning algorithm is required to distinguish among these overlapped samples and identify them properly.

### 4.3. Cancer Image Classification Using CNN

CNN refers to a group of neural networks (NNs) that uses convolution operations to extract features from the input data. Convolution is a mathematical operation that compares two different functions, measures the amount of similarity they have in various regions, and expresses it as an integral. Interestingly, the development of CNN was inspired by the biological brain, especially how the neurons in the visual cortex of the brain process images [46]. CNN is primarily designed to work with 2D and 3D data, but it can easily be customized to the process data of other dimensions. A CNN model’s operations can be divided into two principal stages—feature extraction and classification/regression. The elegance of CNN is that it can automatically extract features from raw data without any prior processing. That been said, an ML model is as good as the data fed to it; hence, employing some preprocessing and noise removal techniques on the data before passing it to the model often helps to elevate its performance [47].

A CNN model is a collection of multiple layers. There are three main types of layers; namely, the convolutional layer, the pooling layer, and the fully-connected layer. A number of small functions, called kernels or filters, are taken and compared with all the images being processed by the model in the convolution layer. This process generates a set of features from each input sample, usually a dimension higher than the input. Since the model extracts these features by itself, they are called automatic features. If we consider a signal $P_{x}$, and convolute it with a kernel ω, the output of that convolution will be as follows:

(12) $$\left(P_{x}*\;\omega \right)\left(\alpha \right)=\int P_{x}\left(t\right)\omega \left(\alpha -t\right)d\alpha$$

where, $\alpha \in R^{n}$ for all n≥1. Here, $P_{x}$ is called the input layer of convolution, and output is called feature-map or activation [48]. Now, in the case of data of higher dimensions, we need to consider t as a discrete parameter and rewrite Equation (12) for a discrete convolution as follows.

(13) $$\left(P_{x}*\;\omega \right)\left(\alpha \right)=\underset{\alpha }{\sum }P_{x}\left(t\right)\omega \left(\alpha -t\right)$$

In this process, α travels overs all the values in the space and is not bounded to a particular dimension. Equation (12) is dimension-independent as well. However, since we are working with 2D data, the output of a 2D convolution on a sample square image, I of m×m pixels, can be expressed as follows:

(14) $$\left(I*\;K\right)\left(i,j\right)=\overset{m-1}{\underset{p=0}{\sum }}\overset{m-1}{\underset{q=0}{\sum }}I\left(p,q\right)K\left(i-p,j-q\right)$$

At the output of a convolution layer, the input image becomes less recognizable. However, certain information such as the edges, orientation, and patterns become more visible, and these are the underlying properties from which machines learn.

A pooling layer’s function is to reduce the size (spatial dimensions) of the feature-map without losing a lot of useful information. Among the variants of pooling options, max-pooling and average-pooling are the most common. Given a small block of the feature-map, max-pooling and average-pooling only keep the maximum value and the average value of that block, respectively, and exclude all the other values. If a max-pooling operation is performed with a kernel size (𝓅×𝓅) and stride of 𝓅 on a (𝒾×𝒿) feature-map, it will result in a $\left(\frac{𝒾}{𝓅}\times \frac{j}{𝓅}\right)$ feature-map.

Depending on the size of the input image and the architecture of the CNN-model, any number of convolution and pooling layers can be applied. However, the feature-map resolution decreases with each new layer, and the machine gets fewer but more relevant features to work with. The fully connected layer is usually the last layer of a CNN model, which performs the classification or regression task following the principles of multi-layer perceptrons. After a series of convolutions and poolings, all the extracted feature-maps are flattened to get a single vector of features. This vector acts as the input of a NN. It is called fully-connected NN because every node of a given layer is connected to all the nodes of the next layer of NN. The NN output is a class label (for classification) or a value (for regression) that the model has decided for a particular sample.

**Figure 7:**
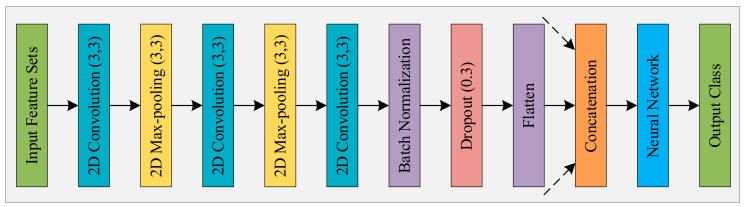
The architecture of the employed CNN model (single channel).

Figure 7 presents the architecture of the CNN model we employed to solve our classification problem. The model has three convolution layers, two max-pooling layers, a batch-normalization layer, and a dropout layer. As stated in the previous section, we selected four sets of features extracted from each image. Each set of features were processed separately by the employed convolution and pooling layers. Their outputs were flattened and concentrated immediately before the fully connected layer. The purpose of this process is to bring diversity in the knowledge extracted by the machine and help it to know the samples more intimately, which, in turn, will enable it to categorize them more accurately.

## 5. Results and Discussion

In this section, we present the acquired results of the performed ML experiment. Table 2 provides further information on the CNN model created to classify the histopathological cancer images. We used 70% of the images (randomly chosen) to train this supervised learning model and the remaining 30% image to test it. Since we are working with a balanced dataset (i.e., each class has the same number of samples), the model will be less prone to bias towards a particular class while making decisions. We will present the model’s performance on both subsets and evaluate it based on the widely used evaluation parameters, including accuracy, precision, recall, F-measure, and confusion matrix of the classification.

**Figure 8:**
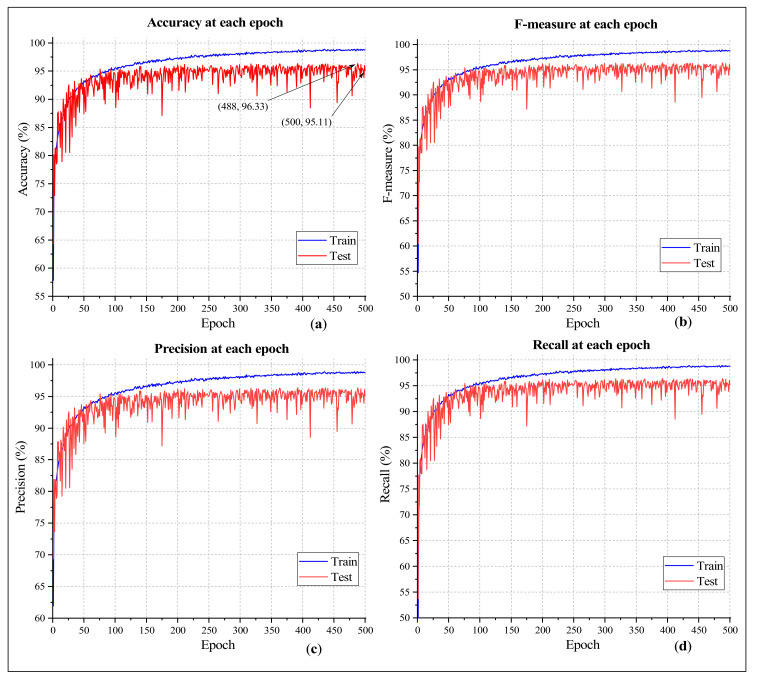
Classification outcome showing (**a**) accuracy, (**b**) F-measure, (**c**) precision, and (**d**) recall at each epoch.

Figure 8a presents the classification accuracy at each epoch of the proposed model. The experiment was carried out for 500 epochs. On the testing subset, the classification accuracy at the last epoch was 95.11%; however, the best outcome was achieved at the 392nd and 488th epoch, both of which yielded an accuracy of 96.33%. As seen in the figure, the training accuracy curve ascended towards the top gradually and almost steadily. The highest training accuracy was 98.91% (493rd epoch), which is very close to the accuracy of the last epoch (98.87%). The testing accuracy curve was not as steady as the training accuracy curve, which indicates the occasional decline in performance. However, the result improved as the training process continued. After the 100th epoch, almost 55% of the testing subset’s recorded accuracy values were over 95%. The curve fell below 90% only four times, which assures that the model can provide a good classification outcome even if it is built with fewer epochs.

The other curves depicted in Figure 8 are the F-measure, precision, and recall curves. Precision yields the positive predictive value (PPV) of a classification, which is the ratio of the correctly identified samples to all the samples that have been positively identified as a particular class. Recall, also known as the sensitivity, indicates the proportion of the positive instances of a specific class that were accurately identified. And finally, F-measure is a harmonic mean of precision and recall, which is a more dependable parameter than the classification accuracy while judging the performance of a classifier. As Figure 8b,d show, all six curves closely follow their corresponding accuracy curves, which indicates that the model’s performance is very reliable, and it is not biased towards any particular class.

**Figure 9:**
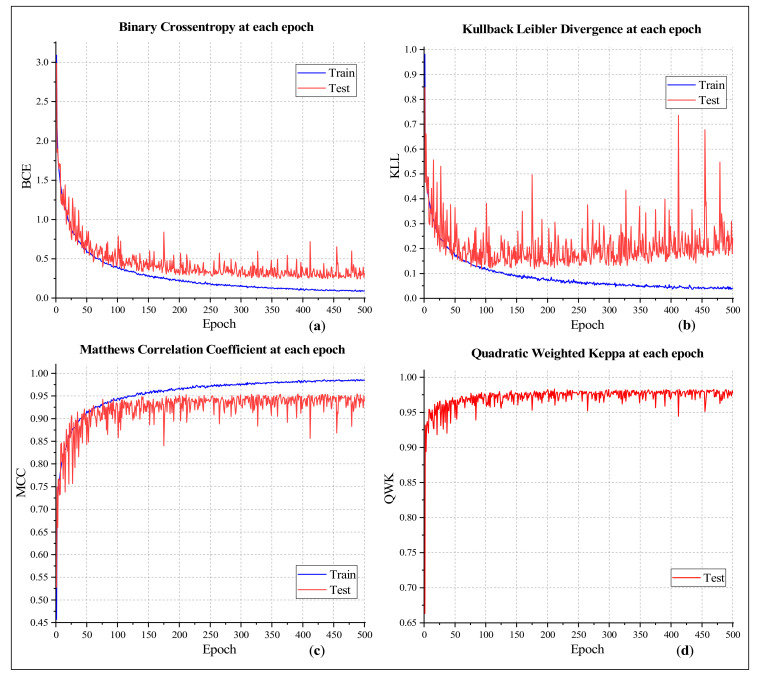
Classification outcome showing (**a**) BCC, (**b**) KLL, (**c**) MCC, and (**d**) QWK values at each epoch.

Figure 9 presents a few more curves to solidify the claimed results. Binary Cross-entropy (BCE) and Kullback Leibler Divergence (KLL) are two parameters that express the amount of information loss in each classification attempt. Figure 9a,b indicate that, as the number of epochs increased, both loss values decreased for the training subset. Results on the testing subset fluctuate rapidly, and the fluctuation is quite similar to the testing accuracy curve. Matthews’s Correlation Coefficient (MCC) is another statistical measure that takes all the four properties of a confusion matrix (i.e., true positives, true negatives, false positives, and false negatives) into account while judging the classification performance. It is considered an even better matric than the F-measure [49]. The highest MCC values acquired in this experiment were 0.9865 and 0.9546 for training and testing classifications, respectively (Figure 8c). Quadratic Weighted Kappa (QWK) is a chance-adjusted matric to judge the reliability of categorical measurements [50]. A QWK value above 0.8 indicates a good agreement between the algorithm’s predictions and a few trusted labels of the same objects. Figure 9d presents the QWK values at each epoch of the classification on the testing subset. It illustrates that almost all the QWK values were above 0.9, which proves the cogency of the proposed method.

**Figure 10:**
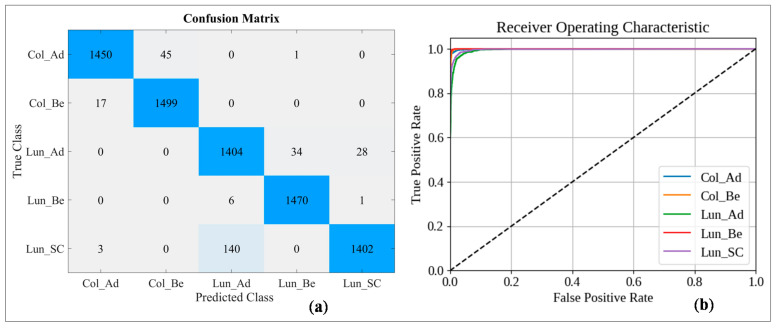
Classification outcome showing the (**a**) confusion matrix and (**b**) ROC curve of the 488th epoch.

Furthermore, in Figure 10, we present the confusion matrix and the Receiver Operating Characteristic (ROC) curves of the classification on the testing subset at the 488th epoch. As seen from the confusion matrix, only 285 samples out of 7500 images were misclassified. The class *Lun_Be* had the best classification outcome; whereas, the class *Lun_Ad* has the highest misclassification rate. These outcomes are also apparent in the ROC curves. The *Col_Ad*, *Col_Be*, and *Lun_Be* curves are almost touching the top-left corner, as the classifier was very successful at distinguishing their samples. However, it faced difficulties while categorizing the samples that belong to the other two classes. Overall, it can be said that the described ML model is highly accurate at identifying these classes, although there remains room for improvement.

Table 3 provides a comparison (in terms of the proposed method’s obtained results) with some of the well-known ML-based lung and colon cancer classification methods discussed in Section 2. However, most of these results are not directly comparable since we worked with a novel dataset that is quite different from the ones used in the cited studies. Nonetheless, they have been put into comparison because the objective remains the same. As seen from the table, our method outperforms most of the other cancer identification methods in terms of the maximum classification accuracy; the only exceptions are the studies described in [19,28,29,31,34]. In the case of [19,29], apart from the accuracy values, only the recall values were presented, and both of them (94% and 93%) are lower than what our model has achieved (96.37%). Among the other studies, the authors of [28] worked with a set of colonoscopy images, and [31,34] worked based on CT scan images, so straightforward comparisons cannot be made. Only the studies cited in [51,52,53] work with the LC25000 dataset. Among them, reference [51] involves only the colon samples of the dataset and reported lower accuracy and F-measure scores than the proposed method. Although [52,53] reported higher accuracy scores, they performed classifications either on the lung samples (three-class classifications) or on the colon samples (binary classifications). As we included all the dataset samples (lung and colon) and performed a five-class classification, the acquired results are not exactly comparable. We hope more studies will be conducted involving the images of this dataset. Nevertheless, based on the discussions provided earlier in this section, it can be concluded that the proposed methods can fulfill the task of lung and colon cancer tissue classification with convenient accuracy and high reliability.

## 6. Conclusions and Future Works

In this paper, we described a novel DL-based supervised learning method that identifies five different types of tissues (three cancerous, two non-cancerous) found in lung and colon tumors by analyzing their corresponding pathological images. We used the LC25000 dataset to train and validate our method. Four sets of features were extracted using two types of domain transformations for image classification. The resultant features were concatenated to build a combined set of features that contains both types of information. The acquired results assure that with a 96.33% peak classification accuracy, the model is highly accurate and reliable (96.38% F-measure score) for lung and colon cancer identification. However, analysis of the results suggests that there is room for improvement in the obtained performance in two of the five classes. A comparison with similar cancer diagnosis methods reveals that the proposed method shows superior performance than most of them. Using this computer-based identification method in the medical centers will allow pathologists to diagnose more lung and colon cancer cases in less effort, cost, and time. In the future, we plan to work on the architecture of the classification model and engineer new sets of features from more histopathological images to elevate its performance.

## Figures and Tables

**Table 1:** Contents of the LC25000 dataset and the assigned class labels.

The Type of Cancer	Class Name	Class ID	Number of Samples
Colon Adenocarcinoma	Col_Ad	0	5000
Colon Benign Tissue	Col_Be	1	5000
Lung Adenocarcinoma	Lun_Ad	2	5000
Lung Benign Tissue	Lun_Be	3	5000
Lung Squamous Cell Carcinoma	Lun_SC	4	5000

**Table 2:** Properties of the employed CNN model.

Variable	Value
Image dimensions	64 × 64
Channels	4
Epochs	500
Batch size	64
Filters	64
2D Convolution layers (size)	3 (3 × 3)
Convolution layer activation	Relu
2D Maxpooling layers (size)	3 (3 × 3)
Dropout	30%
Dense layer activation	Softmax
Compiler optimizer	RMSprop
Compiler loss	Categorical crossentropy

**Table 3:** Comparison of the acquired results with other methods.

Reference	Cancer Type	Image Type	Classifier	Accuracy * (%)	Precision * (%)	Recall * (%)	F-Measure * (%)
[14]	Lung	Biopsy image	mSRC	88.1	84.6	91.3	86.6
[15]	Colon	Histopathological	SVMs	–	73.7	68.2	70.8
[16]	Lung	CT scan	ANN	93.3	–	91.4	–
[18]	Colon	Histopathological	SC-CNN	–	78.3	82.7	80.2
[19]	Colon	Histopathological	RF	99	–	94	–
[20]	Lung	CT scan	MC-CNN	87.14	–	93	–
[21]	Lung	CT scan	CNN	89.9	–	–	–
[22]	Colon	Colonoscopy	AlexNet	91.47		91.76	
[23]	Lung	–	RNN	98	–	96	–
[24]	Lung	CT scan	CNN	92.63	–	90.7	–
[25]	Lung	CT scan	RestNet50 + SVM RBF	93.19	73.48	85.38	78.83
[26]	Lung	CT scan	DFCNet	89.52	–	82.54	–
[27]	Colon	Histopathological	RF	85.3	–	–	85.2
[28]	Colon	Colonoscopy	Faster R-CNN	98.5	100	98.5	99.24
[29]	Colon	Colonoscopy	CNN	96.4	–	93	–
[30]	Colon	Colonoscopy	CNN	90.28	74.34	68.32	71.2
[31]	Lung	CT scan	DAELGNN	99.65	99.67	99.78	99.73
[33]	Lung	CT scan	CNN	93.9	–	93.4	–
[34]	Lung	CT scan	CNN	97.9	98.06	98.07	98.06
[35]	Lung	CT scan	EM	96.2	97.4	98	98.4
[51] ^#^	Colon	Histopathological	RESNET-50	93.91	95.74	96.77	96.26
[52] ^#^	Lung	Histopathological	CNN	97.89	–	–	–
[52] ^#^	Colon	Histopathological	CNN	96.61	–	–	–
[53] ^#^	Lung	Histopathological	CNN	97.2	97.33	97.33	97.33
Proposed	Lung & Colon	Histopathological	CNN	96.33	96.39	96.37	96.38

* best result reported on the study if multiple classifications were performed; — the associated matric was not reported in the corresponding article; ^#^ works with the LC25000 dataset.

## Data Availability

The data that is used in this study is publicly available in https://www.kaggle.com/andrewmvd/lung-and-colon-cancer-histopathological-images.
